# MicroRNAs: New Players in the Pathobiology of Preeclampsia

**DOI:** 10.3389/fcvm.2017.00060

**Published:** 2017-09-25

**Authors:** Kelsey R. Bounds, Valorie L. Chiasson, Lu J. Pan, Sudhiranjan Gupta, Piyali Chatterjee

**Affiliations:** ^1^Department of Internal Medicine, Baylor Scott & White Health, Texas A&M Health Science Center, Temple, TX, United States; ^2^Department of Medical Physiology, Baylor Scott & White Health, Texas A&M Health Science Center, Temple, TX, United States

**Keywords:** pregnancy, microRNAs, preeclampsia, angiogenesis, inflammation

## Abstract

Our understanding of how microRNAs (miRNAs) regulate gene networks and affect different molecular pathways leading to various human pathologies has significantly improved over the years. In contrary, the role of miRNAs in pregnancy-related hypertensive disorders such as preeclampsia (PE) is only beginning to emerge. Recent papers highlight that adverse pregnancy outcomes are associated with aberrant expression of several miRNAs. Presently, efforts are underway to determine the biologic function of these placental miRNAs which can shed light on their contribution to these pregnancy-related disease conditions. The discovery that miRNAs are stable in circulation coupled with the fact that the placenta is capable of releasing them to the circulation in exosomes generates a lot of enthusiasm to use them as biomarkers. In this review, we will summarize the recent findings of our understanding of miRNA regulation in relation to PE, a hypertensive disorder of pregnancy. Particular emphasis will be given to the role of key miRNA molecules such as miR-210 and miR-155 that are known to be consistently dysregulated in women with PE.

## Introduction

Preeclampsia (PE) is a multisystem pregnancy disorder that affects about 10 million women globally (1). PE is known to significantly contribute to maternal and perinatal morbidity and mortality. Each year approximately 76,000 maternal deaths and 500,000 babies die due to hypertensive disorders of pregnancy (2). PE is diagnosed by *de novo* onset of hypertension (≥140 mmHg systolic blood pressure or ≥90 mmHg diastolic blood pressure) at or after midgestation and presence of any one of these symptoms such as proteinuria, thrombocytopenia, renal insufficiency, cerebral, or visual disturbances (3). Because proteinuria does not always correlate with outcomes, it is no longer considered a clinical diagnostic feature of PE (4). The incidence of PE is steadily rising due to additional risk factors that include advanced maternal age, obesity, diabetes, etc. (5). Recent pieces of evidence predict long-term risk of cardiovascular diseases such as stroke, pulmonary hypertension, and metabolic or endocrine problems in both the mother and the child (6). PE can lead to more severe complications such as Hemolysis Elevated Liver enzymes and Low Platelet count (HELLP) syndrome or eclampsia with seizures and visual disturbances. Currently, the only curative therapy of PE is delivery of the baby and the placenta.

The exact underlying causes of PE still remain elusive. PE is thought to occur as a consequence of several factors, including defective spiral artery remodeling, placental oxidative stress, endothelial dysfunction, and systemic inflammation. In normal pregnancies, cytotrophoblasts (CTBs) migrate through the decidua and myometrium in order to invade the maternal spiral arteries of the endothelium and tunica media which supply blood to the developing fetus. This causes the arteries to become large vessels with low resistance facilitating sufficient blood flow to the placenta. In the first stage of PE, the CTBs fail to penetrate the myometrium creating narrow vessels resulting in placental hypoperfusion leading to abnormal remodeling of the spiral arteries (7). The second stage of the disease occurs due to failure of spiral artery remodeling and hypoperfusion leading to placental hypoxia, oxidative stress, endoplasmic reticulum (ER) stress, and subsequent release of several placental factors into the maternal circulation (3). The release of these factors and their interaction with the maternal vasculature results in endothelial dysfunction, a typical characteristic of PE. In addition, these factors also contribute to excessive systemic inflammation and an imbalance of the immune system that causes clinical manifestations of PE.

Regardless of the initiating causes, the placenta is known to play a pivotal role in the initiation and progression of PE. Removal of the placenta causes cessation of clinical symptoms of PE. Therefore, it is important to understand how the gene regulatory networks are controlled within the placenta during PE. In recent years, microRNAs (miRNAs) have garnered much attention as they have emerged as a new class of gene expression regulators. In this review, we will discuss our current understanding of the role of the most consistently dysregulated miRNAs, miR-210 and miR-155 in PE.

## miRNAs and Their Biogenesis

MicroRNAs are small (~22–25 nt), endogenous, single-stranded, non-coding RNAs that regulate gene expression preferentially by binding to the untranslated region (3′UTR) of a target gene (8). These regulatory molecules play an important role in the post-transcriptional regulation of gene expression by causing translational inhibition or mRNA cleavage thereby silencing gene expression unlike protein-coding genes (9). There are about 2,500 miRNAs and 1,000 of these are validated in humans alone (10). One miRNA can bind to multiple targets and result in the translational suppression of numerous genes. miRNAs are involved in several critical processes such as development, cell differentiation, and migration to name a few. Not surprisingly miRNAs also contribute to the conception and maintenance of pregnancy (11) by regulating key processes such as inflammation (12), immune tolerance (13), angiogenesis (14), and apoptosis (15). Aberrant expression of miRNAs is found in several pregnancy-related disorders such as PE, intrauterine growth restriction, and preterm birth.

The miRNA biogenesis occurs *via* multistep process and is regulated by a set of proteins and enzymes at various steps such as transcription, processing steps, or miRNA turnover. miRNAs are first transcribed as long primary transcripts by RNA pol II. miRNA sequences are located within the hairpin structure of the primary transcript. In animals, the nuclear RNase III enzyme, Drosha, cuts the primary miRNA (pri-miRNA) leaving behind about 70 nt hairpin sequence called the precursor miRNA or (pre-miRNA) within the nucleus. Then, exportin-5 exports the pre-miRNA out of the nucleus to be cleaved by Dicer-1, a cytoplasmic RNase III enzyme. This forms a 22-nt-long double-stranded duplex. This duplex then associates with an Argonaute family protein (the core of the RNA-induced silencing complex or RISC). The arm of the duplex that is incorporated into the RISC mediates association with target mRNAs by complementary base pairing. Both strands of the mature miRNA have a potential to bind and regulate their targets, but one strand usually accumulates at significantly higher levels which is the functionally dominant miRNA and the minor product is often preferentially degraded. GW182 then interacts with Ago proteins and promote translational inhibition and mRNA degradation (9, 16). GW182 can act as a scaffold to interact with poly A binding protein (PABP) and competes for binding to PABP with eukaryotic initiation factor 4 G. Due to this interaction, mRNAs are not circularized and this leads to the decrease in translation efficiency. The resulting mRNAs are subsequently targeted for rapid degradation by deadenylase complex (16). This is followed by decapping of the mRNA and makes it prone to exonucleolytic cleavage. The cellular concentration of a miRNA is expected to correlate with its repressive activity. A highly expressed miRNA is thought to repress the translation of its target more and, therefore, be more functionally important than that of an under expressed miRNA. This is supported by several observations of overexpression of miRNAs leading to dose-dependent decreases in the levels of the target mRNAs (17–19).

**Figure 1:**
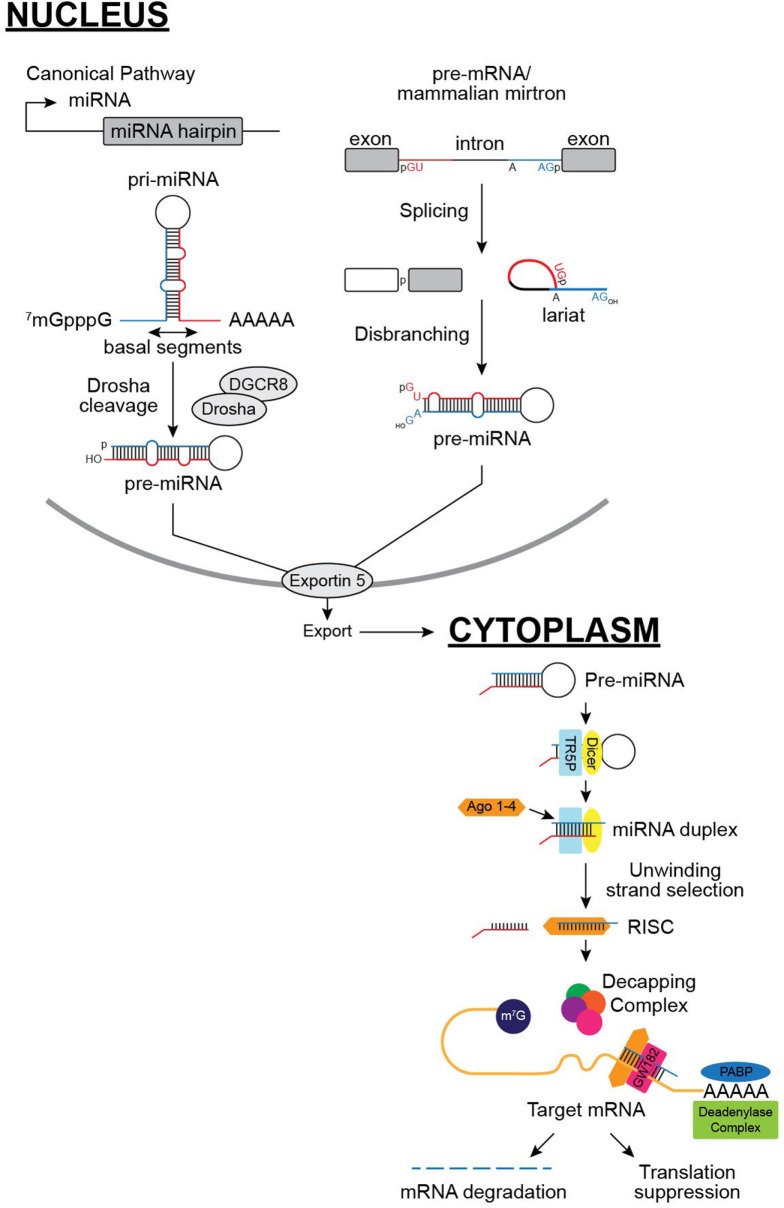
MicroRNA (miRNA) biogenesis pathways: in the canonical pathway, miRNAs are first transcribed by RNA polymerase II in the nucleus that generates a primary miRNA (pri-miRNA), which are then processed by the Drosha–DGCR8 complex to produce precursor miRNAs (pre-miRNAs). Pre-miRNAs are then transported via exportin 5 into the cytoplasm, and are processed by Dicer-TRBP and loaded into AGO2-RISCs to downregulate target gene expression. GW182 (shown here) binds to PABP and prevents circularization of mRNA and promotes rapid degradation. miRNAs are also produced though non-canonical pathways (shown in the right), such as spliceosome-dependent mechanisms, without Drosha cleavage as shown here.

In addition to this canonical pathway, there have been other miRNA pathways recently discovered (Figure 1). Mirtrons, an atypical miRNA, is produced from short hairpin introns that are released by the splicing machinery. Then, they are linearized by the lariat debranching enzyme and further folded into pre-miRNA. Mirtrons bypass Drosha cleavage unlike the canonical pathway. However, they get processed further in a comparable manner (20). Another pathway that has been recently discovered shows direct cleavage of pre-miRNA by Ago2 not Dicer as in the traditional canonical pathway (21). The function of these miRNAs during pregnancy and pregnancy-related disease conditions are currently unknown.

The importance of miRNA biogenesis machinery is highlighted by the observation that the lack of even a single protein in this pathway causes profound defects in different reproductive organs. Drosha (a nuclear RNase III enzyme) is responsible for cleaving pri-miRNAs to pre-miRNAs. If Drosha is specifically knocked down in spermatogenic cells in postnatal testes using Cre-Lox, spermatogenesis is disrupted (22). The role of another protein DGCR8 involved in the miRNA biogenesis pathway that interacts with Drosha to produce pre-miRNA in the nucleus is determined using a conditional knockout mouse model. Kim et al. demonstrated that a Dgcr8 conditional knockout mouse produced using a progesterone receptor (PR)-Cre was required for uterine development and fertility (23). In addition, DGCR8-dependent miRNAs are required for immune modulation, reproductive cycle, and steroid hormone responsiveness. Loss of Dicer (a cytoplasmic RNase III enzyme) leads to embryonic lethality and is essential for maintaining the stem cell population during early mouse development (24). All the key molecules of the miRNA biogenesis pathway have also been found to be expressed in the placenta (25). Ago2 has been shown to be essential for early stages of mouse embryogenesis (26). A mutation in Ago2 in mouse cause defects in placental development and embryonic death at midgestation (27). Whether other miRNA biogenesis proteins also play a critical role in placental development remains unanswered. This is largely due to the fact that a mouse deficient in any of the miRNA biogenesis proteins often exhibit early developmental defects prior to placenta formation.

## Placental miRNAs and Their Function

MicroRNAs are abundantly expressed in the placenta during pregnancy and a study by Liang et al. demonstrated the placental miRNA profiles (28). A major source of placental miRNAs is the villous trophoblasts (29, 30). Mammalian placental miRNAs emerged late during evolution. The placenta expresses miRNAs at different stages of pregnancy, including some that are expressed in a spatio-temporal manner. This suggests that miRNAs play a role in controlling genes and are also modulated by several additional factors such as environment, signaling, and epigenetic pathways.

Interestingly, some novel miRNAs are specifically expressed in the placenta (31). These placenta-specific miRNAs are largely clustered in three groups such as chromosome 14 miRNA cluster (C14MC), C19MC, and miR-371-373 cluster. A large part of trophoblastic miRNAs (trophomiRs) are expressed from a gene cluster C14MC spanning about 40 kb containing 52 miRNAs and is derived exclusively from maternally imprinted genes. The cluster is highly expressed at the first trimester and gradually less expressed in the third trimester. The cluster is mostly expressed in the embryonic and the placental tissues and is known to be involved in regulation of cellular differentiation and fate (31).

Another gene cluster found on chromosome 19, C19MC spans about 100 kb of genomic DNA and harbors 46 intronic miRNA genes that express 58 miRNA species (32). The miRNAs from this one cluster is the most abundant in trophoblasts in the human placenta. For C19MC miRNAs paternally inherited allele is only expressed in the placenta. Within the placenta, the C19MC miRNAs are expressed as early as week 5 of pregnancy and the expression increases as pregnancy advances. Hromadnikova et al. reported an upregulation of circulating C19MC miRNAs (miR-516-5p, miR-517*, miR-520a*, miR-525, and miR526a) in patients with PE (33). Recent findings by Delorme-Axford et al. suggest that high levels of expression of C19MC cluster in primary human trophoblast confer resistance to infection by viruses (34). These miRNAs function by inducing autophagy in placental cells. Interestingly, overexpression of C19MC cluster also confers viral resistance to non-placental cells which strongly indicates that this cluster is important to attenuate invading viral pathogens.

miR-371-373 cluster is a small miRNA cluster in humans and a homologous cluster miR-290-295 is also found in mice. Interestingly, miR-371 cluster is also found on chromosome 19 within 1,050 bp region adjacent to the C19MC cluster. This cluster is highly expressed in the primary trophoblast cells and increased in the third trimester. The cluster is also expressed in early embryonic primordial germ line, the germ line stem cell component of adult testis and stem cell lines from early mouse lineages. A detailed review of how these robustly expressed placenta-specific miRNAs are involved in pregnancy complications can be found in the publications by Morales-Prieto et al. (31) and Mouillet et al. (35).

Given that miRNAs play an important role in several processes, an aberrant placental expression of miRNAs would correlate with several pathological diseases including PE. Recent experimental data suggests that angiogenesis, trophoblast proliferation, and immune tolerance which are key processes in PE are governed by miRNAs. Angiogenesis is known to play a pivotal role in PE pathogenesis and several miRNAs (angiomiRs) are known to alter angiogenic pathways. miR-16 and miR-29 are vascular endothelial growth factor (VEGF)-A and the reduction in VEGF-A subsequently inhibits migration of human umbilical vein endothelial cells (HUVECs) (36–38). miR-494 arrests the transition of growth and synthesis phase (G_1_/S transition) by targeting cyclin-dependant kinase 1 (CDK1) and cyclin D1 (CCND1) (39). The supernatant from miR-494 overexpressing dMSCs impairs HUVEC capillary formation by suppressing VEGF. Furthermore, miR-17, miR-20a, and miR-20b overexpression causes defective cytotrophoblast migration and spiral artery remodeling by targeting Ephrin B2 and Ephrin B4 (37, 40). miR-125b-1-3p, miR-328, and miR-21 are also implicated as angiomiRs. Other angiomiRs such as miR-210 and miR-155 will be discussed in detail later in this review.

Failure of trophoblast invasion leads to defects in spiral artery remodeling, a hallmark of PE pregnancies. Several miRNAs, including miR-16, miR-29b, miR-34a, miR-155, miR-210, and miR-675, decrease proliferation and migration of trophoblasts. Low expression of miR-34a in PE is associated with an increase in SERPINA3 that is involved in trophoblast invasion (41). In contrary, miR-378a-5p, miR-376c, and miR-21 have been shown to enhance trophoblast proliferation and invasion *via* modulating the nodal signaling pathway (42–44). Another well-studied cluster is the miR-17-92 cluster that can regulate the differentiation of primary human trophoblasts (45). However, the deletion of miR-17-92 cluster *in vivo* in mouse gives rise to smaller pups that eventually die at birth (46).

During normal pregnancy, a tolerogenic immune environment is regulated by several immune-regulatory miRNAs. miR-17-92 cluster, miR-146a, miR-155, and miR-223 are known to be dysregulated in PE and associated with many immune cells such as macrophages, dendritic cells, and Tregs function (47). miR-126 also modulates innate immune responses in plasmocytoid DCs (48). Interestingly, high expression of TGFβ3 stimulates miR-494 in decidual mesenchymal stem cells (dMSCs) which in turn inhibits M2 macrophage polarization by reducing PGE2 production (49). miR-181a was found to be upregulated in MSCs of severe PE compared to normal patients and blocks TGFβ pathways (50). HLA-G is known to be involved in developing immune tolerance in pregnancy and miR-152 has been shown to repress HLA-G expression in JEG-3 choriocarcinoma cells. Furthermore, miR-152 plays a role as an immune response enhancer by increasing NK cell-mediated cytolysis (51). miR-152 expression has been found to be higher and conversely, HLA-G expression to be lower in PE. Thus, it is tempting to speculate that miR-152 may play a role in regulating HLA-G expression in PE. Further studies are needed to fully understand the role of miRNAs in immune tolerance during pregnancy. In this review, we will discuss about the role of miR-210 and miR-155 that are consistently seen to be upregulated in PE patients and the important pathways they affect.

## The Role of miR-210 in PE

It has been reported that multiple miRNAs are upregulated or downregulated in placentas/blood/sera/plasma of women with PE compared to normal pregnant women. In particular, one miRNA, miR-210 has been shown to be overexpressed in preeclamptic placentas by several independent groups (52, 53). First, Pineles et al. screened 157 miRNAs using qRTPCR and demonstrated that miR-210 is upregulated in PE placentas. Several subsequent studies by Zhu et al., Mayor-Lynn et al., and Enquobahrie et al., further confirmed increased expression of miR-210 in the placentas of PE women (53–55). miR-210 is an intronic miRNA located within genetic loci of transcript AK123483. Transcription factors that regulate the miR-210 expression include hypoxia-inducible factors (HIF-1α, HIF-2α), and NF-κB (56). While it has been reported that placental miR-210 expression is upregulated in PE patients there are few targets of miR-210 that have been identified in the etiology of PE. A few studies to date are described as follows. miR-210 has been strongly linked with hypoxia that leads to insufficient trophoblast invasion and abnormal spiral artery remodeling contributing to PE (57) and is upregulated by (HIF-1α) which binds to HIF responsive element on the proximal promoter 400 bp upstream (58). By overexpressing miR-210, HIFs control the response to hypoxia on a cellular level by regulating genes involved in different processes such as angiogenesis, erythropoiesis, cell proliferation, differentiation, apoptosis, inflammation, and metabolism.

Zhang and colleagues demonstrated that miR-210 expression is increased in placentas of PE patients and is also rapidly induced by hypoxia in trophoblast cell lines (57). This group found that miR-210 is not only regulated by HIF-1α but also by NF-kB p50. Both ephrin-A3 (EFNA3) and homeobox-A9 (HOXA9) are known for different biological functions such as cell migration and vascular remodeling and development during embryogenesis are direct targets of miR-210. EFNA3 expression is suppressed solely by translational repression, but HOXA9 expression is suppressed by both mRNA degradation and translational repression. The group also found that both targets were downregulated in placentas with PE compared to placentas from control patients. Ectopic expression of miR-210 in trophoblast cells attenuated cell migration and invasion.

One study hypothesized that overexpression of miR-210 would cause mitochondrial dysfunction in PE. When mitochondria are dysfunctional they produce excessive amounts of reactive oxygen species (ROS) which could be a trigger of PE. During normal pregnancies, there is a state of oxidative stress and this is only heightened during pregnancies plagued by PE. ROS, along with estrogen and some inflammatory cytokines, are involved in stabilizing HIF-1α during normoxia. Muralimanoharan et al. found mitochondrial dysfunction in placentas with PE which was associated with elevated ROS production and HIF-1 stabilization. They also found an upregulation of miR-210 and downregulation of ISCU (iron–sulfur cluster scaffold homolog) in PE placentas (59). Their data suggest that the upregulation of miR-210 represses mitochondrial function *via* mitochondria-associated ISCU. Another group evaluated the association between miR-210 and ISCU (60). They found an inverse correlation—when there was an overexpression of miR-210 in their trophoblast cell lines there was a decrease in ISCU. Lee et al. also showed an increase in iron deposition in Swan-71 trophoblasts after hypoxia was induced. They also found an accumulation of intracellular iron and decreased matrigel invasion after inhibiting ISCU through transfection of the Swan-71 trophoblasts which suggests that downregulation of ISCU by miR-210 could inhibit trophoblast invasion, a common precursor to PE. This study is yet another pathophysiological finding that connects miR-210 and PE.

Another study using a high throughput screening (HTS)-based miRNA profiling of human placentas demonstrated miR-210 to be increased in PE patients. A steroidogenic enzyme, hydroxysteroid (17-β) dehydrogenase 1 that is expressed mainly in the syncytiotrophoblasts of chorionic villi of the placenta is a direct target of miR-210. Importantly, plasma levels of hydroxysteroid (17-β) dehydrogenase are mostly reduced prior to onset of PE raising the possibility of using it as a biomarker (61).

Potassium channel modulatory factor 1 (KCMF1) was predicted to be a target of miR-210 using target prediction algorithms. Although other studies indicated that KCMF1 is involved in proliferation, migration, and invasion in epithelial cancers; however, its role in the placenta is unknown. Luo et al. first demonstrated that the levels of KCMF1 were significantly lower in placentas of PE patients and inversely correlate with miR-210 expression (62). KCMF1 is experimentally validated as a direct target of miR-210 using dual luciferase assay in HTR8/SVneo cells. In addition, the inflammatory factor tumor necrosis factor-α (TNF-α) could upregulate miR-210 expression while suppressing KCMF1 levels. Therefore, miR-210 expression may contribute to the occurrence of PE by interfering with KCMF1-mediated signaling in the human placenta.

The role of miR-210 in angiogenesis, iron metabolism, and trophoblast invasion is well established but the role of miR-210 in the regulation of genes related to immune responses is only beginning to emerge. We reported that activation of toll-like receptor 3 (TLR3) *via* poly I:C (a synthetic double-stranded RNA viral mimetic) produces the PE-like symptoms of hypertension, endothelial dysfunction, and proteinuria in mice only when pregnant. We demonstrated that both HIF-1α and NF-κB p50 bind the miR-210 promoter and induce its expression, were also significantly upregulated in poly I:C treated mouse placentas. Poly I:C treatment of human CTBs significantly increased HIF-1α, NF-κB p50, and miR-210 levels. In addition, we demonstrate that miR-210 directly targets signal transducer and activator of transcription 6 (STAT6) resulting in decreases of IL-4 production which causes the balance of pro to anti-inflammatory cytokine production in favor of pro-inflammatory cytokines. These findings demonstrate that in a TLR3-induced preeclamptic mouse model, placental miR-210 expression is induced *via* HIF-1α and NF-κB p50 which may contribute to the development of PE (63). These studies taken together suggest that the multitude of miR-210 functions that affect different pathways during PE, such as mitochondrial dysfunction, angiogenesis, and immune system.

## The Role of miR-155 in PE

Another miRNA that is linked to PE is miR-155 (64–66). It is processed in humans from exon 3 of the non-protein coding B-cell integration cluster (BIC) RNA (67). Its expression is induced in activated B-cells, T-cells, and macrophages and several studies have found it overexpressed in several types of B-cell lymphoma (24).

Zhang et al. reported that overexpression of miR-155 contributes to PE by downregulating cysteine-rich protein 61 (CYR61), an important angiogenic regulating factor during pregnancy (68). A targeted knockout of the CYR61 gene in mice resulted in embryonic death due to placental vascular insufficiency and compromised vessel integrity (69). It has been shown in previous studies that CYR61 gene was significantly decreased in human placentas with PE (70). CYR61 is known to induce the expression of VEGF and this group hypothesized that a decrease in CYR61 would cause a decrease in VEGF levels in placentas with PE. miR-155 targeted a region within the 3′UTR sequence of the CYR61 gene which leads to decreased levels of CYR61 in PE placentas. These findings propose a miR-155-CYR61-VEGF pathway where an overexpression of miR-155 causes a downregulation of CYR61 which leads to decreased levels of VEGF, therefore, reducing placental angiogenesis.

Liu et al. demonstrated that the placental expression of miR-155 increased and vascular endothelial growth factor (VEGF) decreased in a rat model of PE induced by l-nitroarginine methyl ester (l-NAME) (71). Since VEGF plays an important role in the vascular lumen formation during angiogenesis it seems to be a key player in pregnancy. A decrease in VEGF causes reduced angiogenesis which is linked with PE because of improper placental vascular network formation and function such as shallow invasion of trophoblast cells causing placental ischemia. This group also found that the upregulation of miR-155 inversely correlated with a decrease in VEGF levels within the placentas suggesting that miR-155 induces a downregulation of VEGF expression which contributes to the development of PE.

Several researchers have shown miR-155 to attenuate trophoblast proliferation through regulation of cyclin D1 in HTR-8/SVneo extravillous trophoblast cells. It also targets interleukin-1 receptor-associated kinase M (IRAKM), NF-kB inhibitor interacting Ras-like 1 (NKIRAS1), and phosphatase and tensin homolog (PTEN). By targeting these regulators, miR-155 enhances AP-1/NF-kB inflammatory pathways (72). Also, increased expression of miR-155 has been implicated in shallow placental invasion as seen in PE (73).

miR-155 also regulates angiotensin II type 1 receptor expression in umbilical vein endothelial cells from women with severe PE (74). Angiotensin II (Ang II) could play an important role in the pathogenesis of PE by inducing low-grade inflammation on endothelial cells, vascular cells, smooth muscle cells, and immune cells. miR-155 has been also linked with regulating the expression of renin–angiotensin system factors. In this study, Cheng et al. isolated HUVECs from normal pregnant women and patients with severe PE. Furthermore, significant increases in Ang II and AT1R within the HUVECs and reduced expression of miR-155 from PE patients compared to normal pregnant women indicates an association between them. In addition, the authors demonstrate that miR-155 directly targets AT1R. These data suggest that patients with PE have a decreased expression of miR-155 which leads to an increase in AT1R, thus influencing renin–angiotensin system expression contributing to the development of PE.

O’Connell et al. found that miR-155 is a common target of inflammatory mediators, and since it is known to be an oncogene, their findings potentially identified a link between inflammation and cancer (75). In their study, they found that miR-155 was induced by TLRs in macrophages. This means that miR-155 is a component of the primary macrophage response to different types of inflammatory mediators. miR-155 is known to be upregulated in placentas of patients with PE, and previous studies have shown a potential association between miR-155 and PE due to inflammation at the maternal–fetal interface (68). In support, recently both miR-155 and IL-17A were found to be upregulated in late onset PE placentas and serum (76). Table 1 summarizes the placenta-associated miRNAs that are dysregulated during PE.

**Table 1:** A list of placenta-associated microRNAs (miRNAs) involved in preeclampsia (PE).

Function	miRNAs involved	Known targets/pathways
Angiogenesis	miR-16, miR-29	Vascular endothelial growth factor (VEGF)-A
	miR-494	CDK6/CYCD1
	miR-17 miR-20a, miR-20b	Ephrin B2, B4
	miR-125b-1-3p	S1PR
	miR-155	CYR 6, VEGF-A
	miR-21	PTEN, positive regulator of VEGF-A and HIF-1α
	miR-210	EFNA3, HOXA9, HSD17

Trophoblast proliferation	miR-16, miR-29b	Inhibits trophoblast proliferation
	miR-34a	SERPINA3
	miR-210	KCMF-1
	miR-155	CYCD1
	miR-378a-5p, miR-376c, miR-21	Promotes trophoblast proliferation by nodal signaling pathway
	miR-17-92 cluster	Differentiation of primary trophoblasts

Inflammation	miR-146a	Inflammatory pathway
	miR-155	IL-17A pathway
	miR-494	Macrophage proliferation by reducing PGE2 production
	miR-181a	TGFβ pathway
	miR-152	HLA-G
	miR-210	STAT6/IL-4 pathway

In the future, how these miRNAs contribute to endothelial dysfunction and hypertension during pregnancy leading to PE needs to be addressed (77). Specifically, to delineate the role of individual miRNAs in PE additional *in vivo* studies utilizing placenta-specific overexpression or gene knockout models are needed to address these unanswered questions.

## Conclusion

The discovery of miRNAs expanded our knowledge about how they regulate gene expression by modulating several key cellular processes. Currently, research is underway to use miRNA-based biomarkers as diagnostic tool for various diseases including PE. A large number of studies have been performed using miRNA profiling coupled with validation studies of these miRNAs in blood/serum/plasma of PE patients. In the future, the success of using these miRNAs as biomarkers in PE will greatly rely on the determination of expression of these miRNAs at different stages of pregnancy and how it can alter the power of predicting disease progression as well as clinical outcome. Another diagnostic approach gaining attention is the determination of concentration and miRNA content of exosomes across gestation which may serve as an early marker for PE (78). In addition, elucidating the functional role these dysregulated miRNAs play may identify important pathways involved in PE.

A paucity of data of the role of miRNAs in PE presently limits their use in therapeutics. Few miRNAs involved in other diseases are validated in clinically relevant animal models and are presently in preclinical/clinical trials. Typically, in a disease setting, antagomiRs decrease detrimental miRNAs and in contrast miRNA mimics can increase the abundance of beneficial miRNAs. Clinical trials are now underway for a liver-specific miRNA, miR-122 for treatment of chronic HCV infection following studies in mice and non-human primates. The success of administration of anti-miR-122 was mainly due to the ease by which these miRNAs predominantly accumulate in the liver (79). Based on tumor suppressive effects of miR-34, liposome formulated mimic-based drug is also administered currently in patients with primary liver cancer (80). We are in need of appropriate animal disease models and human function studies for future clinical trials using miRNA-based therapeutics.

To facilitate miRNA-based therapeutics in pregnancy-related disorders, future studies with more emphasis on minimizing off target effects, improving timing and dosing to obtain minimal side effects and elucidating the exact uptake mechanism of these miRNAs by other organs are needed. Therefore, increasing our knowledge of the function of miRNAs in pregnancy-related disorders is necessary in order to develop therapeutical strategies in the future.

## Author Contributions

KB, VC, LP, and SG wrote parts of the manuscript and edited. PC wrote, organized, and finalized the manuscript.

## Conflict of Interest Statement

The authors declare that the research was conducted in the absence of any commercial or financial relationships that could be construed as a potential conflict of interest.

## References

[1] Duley L. The global impact of pre-eclampsia and eclampsia. Semin Perinatol (2009) 33(3):130–7. 10.1053/j.semperi.2009.02.010 19464502

[2] Khowaja AR Mitton C Bryan S Magee LA Bhutta ZA von Dadelszen P. Economic evaluation of Community Level Interventions for Pre-eclampsia (CLIP) in South Asian and African countries: a study protocol. Implement Sci (2015) 10:76. 10.1186/s13012-015-0266-5 26007682 PMC4446068

[3] Roberts JM Hubel CA. The two stage model of preeclampsia: variations on the theme. Placenta (2009) 30(Suppl A):S32–7. 10.1016/j.placenta.2008.11.009 19070896 PMC2680383

[4] Leeman L Dresang LT Fontaine P. Hypertensive disorders of pregnancy. Am Fam Physician (2016) 93(2):121–7. 26926408

[5] Bounds KR Newell-Rogers MK Mitchell BM. Four pathways involving innate immunity in the pathogenesis of preeclampsia. Front Cardiovasc Med (2015) 2:20. 10.3389/fcvm.2015.00020 26664892 PMC4671354

[6] Davis EF Lazdam M Lewandowski AJ Worton SA Kelly B Kenworthy Y Cardiovascular risk factors in children and young adults born to preeclamptic pregnancies: a systematic review. Pediatrics (2012) 129(6):e1552–61. 10.1542/peds.2011-3093 22614768

[7] Burke SD Karumanchi SA Spiral artery remodeling in preeclampsia revisited. Hypertension (2013) 62(6):1013–4. 10.1161/HYPERTENSIONAHA.113.02049 24144648

[8] Krek A Grun D Poy MN Wolf R Rosenberg L Epstein EJ Combinatorial microRNA target predictions. Nat Genet (2005) 37(5):495–500. 10.1038/ng1536 15806104

[9] Bartel DP. MicroRNAs: genomics, biogenesis, mechanism, and function. Cell (2004) 116(2):281–97. 10.1016/S0092-8674(04)00045-5 14744438

[10] Friedlander MR Lizano E Houben AJ Bezdan D Banez-Coronel M Kudla G Evidence for the biogenesis of more than 1,000 novel human microRNAs. Genome Biol (2014) 15(4):R57. 10.1186/gb-2014-15-4-r57 24708865 PMC4054668

[11] Robertson SA Zhang B Chan HY Sharkey DJ Barry SC Fullston T MicroRNA regulation of immune events at conception. Mol Reprod Dev (2017). 10.1002/mrd.22823 28452160

[12] Veit TD Chies JA Tolerance versus immune response – microRNAs as important elements in the regulation of the HLA-G gene expression. Transpl Immunol (2009) 20(4):229–31. 10.1016/j.trim.2008.11.001 19038339

[13] Schjenken JE Zhang B Chan HY Sharkey DJ Fullston T Robertson SA. miRNA regulation of immune tolerance in early pregnancy. Am J Reprod Immunol (2016) 75(3):272–80. 10.1111/aji.12490 26804209

[14] Santa LM Teshima LY Forero JV Giraldo AO. AngiomiRs: potential biomarkers of pregnancy’s vascular pathologies. J Pregnancy (2015) 2015:320386. 10.1155/2015/320386 26550492 PMC4621355

[15] Lycoudi A Mavreli D Mavrou A Papantoniou N Kolialexi A. miRNAs in pregnancy-related complications. Expert Rev Mol Diagn (2015) 15(8):999–1010. 10.1586/14737159.2015.1053468 26051307

[16] Wu L Fan J Belasco JG. MicroRNAs direct rapid deadenylation of mRNA. Proc Natl Acad Sci U S A (2006) 103(11):4034–9. 10.1073/pnas.0510928103 16495412 PMC1449641

[17] Shu J Xia Z Li L Liang ET Slipek N Shen D Dose-dependent differential mRNA target selection and regulation by let-7a-7f and miR-17-92 cluster microRNAs. RNA Biol (2012) 9(10):1275–87. 10.4161/rna.21998 22995834 PMC3583858

[18] Wang B Zou A Ma L Chen X Wang L Zeng X miR-455 inhibits breast cancer cell proliferation through targeting CDK14. Eur J Pharmacol (2017) 807:138–43. 10.1016/j.ejphar.2017.03.016 28300591

[19] Wang X Xu X Wang W Yu Z Wen L He K MicroRNA-30a-5p promotes replication of porcine circovirus type 2 through enhancing autophagy by targeting 14-3-3. Arch Virol (2017) 162(9):2643–54. 10.1007/s00705-017-3400-7 28530014

[20] Berezikov E Chung WJ Willis J Cuppen E Lai EC Mammalian mirtron genes. Mol Cell (2007) 28(2):328–36. 10.1016/j.molcel.2007.09.028 17964270 PMC2763384

[21] Ha M Kim VN Regulation of microRNA biogenesis. Nat Rev Mol Cell Biol (2014) 15(8):509–24. 10.1038/nrm3838 25027649

[22] Wu Q Song R Ortogero N Zheng H Evanoff R Small CL The RNase III enzyme DROSHA is essential for microRNA production and spermatogenesis. J Biol Chem (2012) 287(30):25173–90. 10.1074/jbc.M112.362053 22665486 PMC3408133

[23] Kim YS Kim HR Kim H Yang SC Park M Yoon JA Deficiency in DGCR8-dependent canonical microRNAs causes infertility due to multiple abnormalities during uterine development in mice. Sci Rep (2016) 6:20242. 10.1038/srep20242 26833131 PMC4735737

[24] Bernstein E Kim SY Carmell MA Murchison EP Alcorn H Li MZ Dicer is essential for mouse development. Nat Genet (2003) 35(3):215–7. 10.1038/ng1253 14528307

[25] Mouillet JF Chu T Sadovsky Y. Expression patterns of placental microRNAs. Birth Defects Res A Clin Mol Teratol (2011) 91(8):737–43. 10.1002/bdra.20782 21425434 PMC5030720

[26] Lykke-Andersen K Gilchrist MJ Grabarek JB Das P Miska E Zernicka-Goetz M. Maternal Argonaute 2 is essential for early mouse development at the maternal-zygotic transition. Mol Biol Cell (2008) 19(10):4383–92. 10.1091/mbc.E08-02-0219 18701707 PMC2555945

[27] Cheloufi S Dos Santos CO Chong MM Hannon GJ A dicer-independent miRNA biogenesis pathway that requires Ago catalysis. Nature (2010) 465(7298):584–9. 10.1038/nature09092 20424607 PMC2995450

[28] Liang Y Ridzon D Wong L Chen C. Characterization of microRNA expression profiles in normal human tissues. BMC Genomics (2007) 8:166. 10.1186/1471-2164-8-166 17565689 PMC1904203

[29] Morales-Prieto DM Chaiwangyen W Ospina-Prieto S Schneider U Herrmann J Gruhn B MicroRNA expression profiles of trophoblastic cells. Placenta (2012) 33(9):725–34. 10.1016/j.placenta.2012.05.009 22721760

[30] Donker RB Mouillet JF Chu T Hubel CA Stolz DB Morelli AE The expression profile of C19MC microRNAs in primary human trophoblast cells and exosomes. Mol Hum Reprod (2012) 18(8):417–24. 10.1093/molehr/gas013 22383544 PMC3389496

[31] Morales-Prieto DM Ospina-Prieto S Chaiwangyen W Schoenleben M Markert UR. Pregnancy-associated miRNA-clusters. J Reprod Immunol (2013) 97(1):51–61. 10.1016/j.jri.2012.11.001 23432872

[32] Bentwich I Avniel A Karov Y Aharonov R Gilad S Barad O Identification of hundreds of conserved and nonconserved human microRNAs. Nat Genet (2005) 37(7):766–70. 10.1038/ng1590 15965474

[33] Hromadnikova I Kotlabova K Ondrackova M Kestlerova A Novotna V Hympanova L Circulating C19MC microRNAs in preeclampsia, gestational hypertension, and fetal growth restriction. Mediators Inflamm (2013) 2013:186041. 10.1155/2013/186041 24347821 PMC3848305

[34] Delorme-Axford E Donker RB Mouillet JF Chu T Bayer A Ouyang Y Human placental trophoblasts confer viral resistance to recipient cells. Proc Natl Acad Sci U S A (2013) 110(29):12048–53. 10.1073/pnas.1304718110 23818581 PMC3718097

[35] Mouillet JF Ouyang Y Coyne CB Sadovsky Y MicroRNAs in placental health and disease. Am J Obstet Gynecol (2015) 213(4 Suppl):S163–72. 10.1016/j.ajog.2015.05.057 26428496 PMC4592520

[36] Hu Y Li P Hao S Liu L Zhao J Hou Y. Differential expression of microRNAs in the placentae of Chinese patients with severe pre-eclampsia. Clin Chem Lab Med (2009) 47(8):923–9. 10.1515/CCLM.2009.228 19642860

[37] Wang W Feng L Zhang H Hachy S Satohisa S Laurent LC Preeclampsia up-regulates angiogenesis-associated microRNA (i.e., miR-17, -20a, and -20b) that target ephrin-B2 and EPHB4 in human placenta. J Clin Endocrinol Metab (2012) 97(6):E1051–9. 10.1210/jc.2011-3131 22438230 PMC3387422

[38] Li H Ge Q Guo L Lu Z. Maternal plasma miRNAs expression in preeclamptic pregnancies. Biomed Res Int (2013) 2013:970265. 10.1155/2013/970265 24195082 PMC3781840

[39] Chen S Zhao G Miao H Tang R Song Y Hu Y MicroRNA-494 inhibits the growth and angiogenesis-regulating potential of mesenchymal stem cells. FEBS Lett (2015) 589(6):710–7. 10.1016/j.febslet.2015.01.038 25660325

[40] Wang Y Zhang Y Wang H Wang J Zhang Y Wang Y Aberrantly up-regulated miR-20a in pre-eclampsic placenta compromised the proliferative and invasive behaviors of trophoblast cells by targeting forkhead box protein A1. Int J Biol Sci (2014) 10(9):973–82. 10.7150/ijbs.9088 25210495 PMC4159688

[41] Chelbi ST Wilson ML Veillard AC Ingles SA Zhang J Mondon F Genetic and epigenetic mechanisms collaborate to control SERPINA3 expression and its association with placental diseases. Hum Mol Genet (2012) 21(9):1968–78. 10.1093/hmg/dds006 22246292

[42] Luo L Ye G Nadeem L Fu G Yang BB Honarparvar E MicroRNA-378a-5p promotes trophoblast cell survival, migration and invasion by targeting Nodal. J Cell Sci (2012) 125(Pt 13):3124–32. 10.1242/jcs.096412 22454525

[43] Chaiwangyen W Ospina-Prieto S Photini SM Schleussner E Markert UR Morales-Prieto DM. Dissimilar microRNA-21 functions and targets in trophoblastic cell lines of different origin. Int J Biochem Cell Biol (2015) 68:187–96. 10.1016/j.biocel.2015.08.018 26320576

[44] Fu G Ye G Nadeem L Ji L Manchanda T Wang Y MicroRNA-376c impairs transforming growth factor-beta and nodal signaling to promote trophoblast cell proliferation and invasion. Hypertension (2013) 61(4):864–72. 10.1161/HYPERTENSIONAHA.111.203489 23424236

[45] Kumar P Luo Y Tudela C Alexander JM Mendelson CR. The c-Myc-regulated microRNA-17~92 (miR-17~92) and miR-106a~363 clusters target hCYP19A1 and hGCM1 to inhibit human trophoblast differentiation. Mol Cell Biol (2013) 33(9):1782–96. 10.1128/MCB.01228-12 23438603 PMC3624183

[46] Concepcion CP Bonetti C Ventura A. The microRNA-17-92 family of microRNA clusters in development and disease. Cancer J (2012) 18(3):262–7. 10.1097/PPO.0b013e318258b60a 22647363 PMC3592780

[47] Baltimore D Boldin MP O’Connell RM Rao DS Taganov KD MicroRNAs: new regulators of immune cell development and function. Nat Immunol (2008) 9(8):839–45. 10.1038/ni.f.209 18645592

[48] Ferretti C La Cava A miR-126, a new modulator of innate immunity. Cell Mol Immunol (2014) 11(3):215–7. 10.1038/cmi.2014.5 24531618 PMC4085494

[49] Zhao G Miao H Li X Chen S Hu Y Wang Z TGF-beta3-induced miR-494 inhibits macrophage polarization via suppressing PGE2 secretion in mesenchymal stem cells. FEBS Lett (2016) 590(11):1602–13. 10.1002/1873-3468.12200 27149081

[50] Liu L Wang Y Fan H Zhao X Liu D Hu Y MicroRNA-181a regulates local immune balance by inhibiting proliferation and immunosuppressive properties of mesenchymal stem cells. Stem Cells (2012) 30(8):1756–70. 10.1002/stem.1156 22714950

[51] Zhu XM Han T Wang XH Li YH Yang HG Luo YN Overexpression of miR-152 leads to reduced expression of human leukocyte antigen-G and increased natural killer cell mediated cytolysis in JEG-3 cells. Am J Obstet Gynecol (2010) 202(6):592.e1–7. 10.1016/j.ajog.2010.03.002 20430358

[52] Pineles BL Romero R Montenegro D Tarca AL Han YM Kim YM Distinct subsets of microRNAs are expressed differentially in the human placentas of patients with preeclampsia. Am J Obstet Gynecol (2007) 196(3):261.e1–6. 10.1016/j.ajog.2007.01.008 17346547

[53] Zhu XM Han T Sargent IL Yin GW Yao YQ. Differential expression profile of microRNAs in human placentas from preeclamptic pregnancies vs normal pregnancies. Am J Obstet Gynecol (2009) 200(6):661.e1–7. 10.1016/j.ajog.2008.12.045 19285651

[54] Mayor-Lynn K Toloubeydokhti T Cruz AC Chegini N. Expression profile of microRNAs and mRNAs in human placentas from pregnancies complicated by preeclampsia and preterm labor. Reprod Sci (2011) 18(1):46–56. 10.1177/1933719110374115 21079238 PMC3343068

[55] Enquobahrie DA Abetew DF Sorensen TK Willoughby D Chidambaram K Williams MA Placental microRNA expression in pregnancies complicated by preeclampsia. Am J Obstet Gynecol (2011) 204(2):178.e12–21. 10.1016/j.ajog.2010.09.004 PMC3040986 21093846

[56] Chan YC Banerjee J Choi SY Sen CK. miR-210: the master hypoxamir. Microcirculation (2012) 19(3):215–23. 10.1111/j.1549-8719.2011.00154.x 22171547 PMC3399423

[57] Zhang Y Fei M Xue G Zhou Q Jia Y Li L Elevated levels of hypoxia-inducible microRNA-210 in pre-eclampsia: new insights into molecular mechanisms for the disease. J Cell Mol Med (2012) 16(2):249–59. 10.1111/j.1582-4934.2011.01291.x 21388517 PMC3823289

[58] Huang X Le QT Giaccia AJ miR-210 – micromanager of the hypoxia pathway. Trends Mol Med (2010) 16(5):230–7. 10.1016/j.molmed.2010.03.004 20434954 PMC3408219

[59] Muralimanoharan S Maloyan A Mele J Guo C Myatt LG Myatt L. miR-210 modulates mitochondrial respiration in placenta with preeclampsia. Placenta (2012) 33(10):816–23. 10.1016/j.placenta.2012.07.002 22840297 PMC3439551

[60] Lee DC Romero R Kim JS Tarca AL Montenegro D Pineles BL miR-210 targets iron-sulfur cluster scaffold homologue in human trophoblast cell lines: siderosis of interstitial trophoblasts as a novel pathology of preterm preeclampsia and small-for-gestational-age pregnancies. Am J Pathol (2011) 179(2):590–602. 10.1016/j.ajpath.2011.04.035 21801864 PMC3160082

[61] Ishibashi O Ohkuchi A Ali MM Kurashina R Luo SS Ishikawa T Hydroxysteroid (17-beta) dehydrogenase 1 is dysregulated by miR-210 and miR-518c that are aberrantly expressed in preeclamptic placentas: a novel marker for predicting preeclampsia. Hypertension (2012) 59(2):265–73. 10.1161/HYPERTENSIONAHA.111.180232 22203747

[62] Luo R Shao X Xu P Liu Y Wang Y Zhao Y MicroRNA-210 contributes to preeclampsia by downregulating potassium channel modulatory factor 1. Hypertension (2014) 64(4):839–45. 10.1161/HYPERTENSIONAHA.114.03530 24980667

[63] Kopriva SE Chiasson VL Mitchell BM Chatterjee P. TLR3-induced placental miR-210 down-regulates the STAT6/interleukin-4 pathway. PLoS One (2013) 8(7):e67760. 10.1371/journal.pone.0067760 23844087 PMC3699491

[64] Lazar L Rigo J. PP088. The role of microRNA in pathogenesis of preeclampsia-miRNA network analysis. Pregnancy Hypertens (2013) 3(2):99. 10.1016/j.preghy.2013.04.113 26105941

[65] Murphy MS Casselman RC Tayade C Smith GN. Differential expression of plasma microRNA in preeclamptic patients at delivery and 1 year postpartum. Am J Obstet Gynecol (2015) 213(3):367.e1–9. 10.1016/j.ajog.2015.05.013 25981845

[66] Hromadnikova I Kotlabova K Hympanova L Krofta L. Cardiovascular and cerebrovascular disease associated microRNAs are dysregulated in placental tissues affected with gestational hypertension, preeclampsia and intrauterine growth restriction. PLoS One (2015) 10(9):e0138383. 10.1371/journal.pone.0138383 26394310 PMC4579085

[67] Elton TS Selemon H Elton SM Parinandi NL. Regulation of the miR155 host gene in physiological and pathological processes. Gene (2013) 532(1):1–12. 10.1016/j.gene.2012.12.009 23246696

[68] Zhang Y Diao Z Su L Sun H Li R Cui H MicroRNA-155 contributes to preeclampsia by down-regulating CYR61. Am J Obstet Gynecol (2010) 202(5):466.e1–7. 10.1016/j.ajog.2010.01.057 20452491

[69] Mo FE Muntean AG Chen CC Stolz DB Watkins SC Lau LF. CYR61 (CCN1) is essential for placental development and vascular integrity. Mol Cell Biol (2002) 22(24):8709–20. 10.1128/MCB.22.24.8709-8720.2002 12446788 PMC139880

[70] Gellhaus A Schmidt M Dunk C Lye SJ Kimmig R Winterhager E. Decreased expression of the angiogenic regulators CYR61 (CCN1) and NOV (CCN3) in human placenta is associated with pre-eclampsia. Mol Hum Reprod (2006) 12(6):389–99. 10.1093/molehr/gal044 16675545

[71] Liu Q Yang J. Expression and significance of miR155 and vascular endothelial growth factor in placenta of rats with preeclampsia. Int J Clin Exp Med (2015) 8(9):15731–7. 26629069 PMC4658958

[72] Xue P Zheng M Diao Z Shen L Liu M Gong P miR-155* mediates suppressive effect of PTEN 3’-untranslated region on AP-1/NF-kappaB pathway in HTR-8/SVneo cells. Placenta (2013) 34(8):650–6. 10.1016/j.placenta.2013.04.015 23684381

[73] Dai Y Diao Z Sun H Li R Qiu Z Hu Y. MicroRNA-155 is involved in the remodelling of human-trophoblast-derived HTR-8/SVneo cells induced by lipopolysaccharides. Hum Reprod (2011) 26(7):1882–91. 10.1093/humrep/der118 21515911

[74] Cheng W Liu T Jiang F Liu C Zhao X Gao Y microRNA-155 regulates angiotensin II type 1 receptor expression in umbilical vein endothelial cells from severely pre-eclamptic pregnant women. Int J Mol Med (2011) 27(3):393–9. 10.3892/ijmm.2011.598 21234519

[75] O’Connell RM Taganov KD Boldin MP Cheng G Baltimore D. MicroRNA-155 is induced during the macrophage inflammatory response. Proc Natl Acad Sci U S A (2007) 104(5):1604–9. 10.1073/pnas.0610731104 17242365 PMC1780072

[76] Yang X Zhang J Ding Y. Association of microRNA-155, interleukin 17A, and proteinuria in preeclampsia. Medicine (2017) 96(18):e6509. 10.1097/MD.0000000000006509 28471953 PMC5419899

[77] Santulli G. MicroRNAs and endothelial (Dys) function. J Cell Physiol (2016) 231(8):1638–44. 10.1002/jcp.25276 26627535 PMC4871250

[78] Salomon C Guanzon D Scholz-Romero K Longo S Correa P Illanes SE Placental exosomes as early biomarker of preeclampsia – potential role of exosomal microRNAs across gestation. J Clin Endocrinol Metab (2017) 102:3182–94. 10.1210/jc.2017-00672 28531338

[79] Jopling CL. Targeting microRNA-122 to treat hepatitis C virus infection. Viruses (2010) 2(7):1382–93. 10.3390/v2071382 21994685 PMC3185717

[80] Bouchie A First microRNA mimic enters clinic. Nat Biotechnol (2013) 31(7):577 10.1038/nbt0713-577 23839128

